# The Efficacy and Safety of Intra-articular Low Molecular Weight Fraction of Human Serum Albumin for the Management of Moderate to Moderately Severe Knee Osteoarthritis: A Systematic Review and Meta-Analysis

**DOI:** 10.7759/cureus.41240

**Published:** 2023-06-30

**Authors:** Mohammad H Nooh, Mohammed S Alshehri, Ziyad S Alzahrani, Hatem M Alsolami, Amal O Almutairi, Abdulaziz S AlOtaibi, Abdulaziz N Aljohani

**Affiliations:** 1 Medicine, King Saud Bin Abdulaziz University for Health Sciences, Jeddah, SAU; 2 Medical Research, King Abdullah International Medical Research Center, Jeddah, SAU; 3 Orthopaedic Surgery, King Abdulaziz Medical City, Jeddah, SAU; 4 Orthopaedic Surgery, King Saud Bin Abdulaziz University for Health Sciences, Jeddah, SAU; 5 Medicine, King Abdulaziz University, Jeddah, SAU

**Keywords:** intra-articular injection, cartilage, knee joint, osteoarthritis, lmwf-5a

## Abstract

Osteoarthritis is a chronic degenerative joint disease that affects weight-bearing joints. Low molecular weight fraction of 5% (LMWF-5A) human serum albumin is an intra-articular injection that emerged for the treatment of knee osteoarthritis. The aim of this review is to assess the efficacy and safety of LMWF-5A versus placebo through a systematic review and meta-analysis.

The Cochrane Central Register of Controlled Trials (CENTRAL), Medical Literature Analysis and Retrieval System Online (MEDLINE), EBSCO, and ClinicalTrials.gov registry databases were utilized to search for studies. Only randomized controlled trials (RCTs) that evaluated the efficacy of LMWF-5A versus placebo were included. Efficacy endpoints were represented by Western Ontario and McMaster Universities Arthritis Index (WOMAC) A and C scores for pain and function, respectively. Serious adverse events (SAEs), non-serious adverse events (NSAEs), and mortality rates were used to evaluate the safety of the drug. The revised Cochrane risk of bias tool was used for the risk of bias assessment. Seven RCTs (n=2939) that met the inclusion criteria were included.

The meta-analysis did not find significant improvement in pain (WOMAC A) (standardized mean difference (SMD)= -0.01, 95% confidence interval (CI) -0.10 - 0.09, P=0.87, I²=30%). Additionally, no significant change in function was noted (WOMAC C) (SMD=0.01, 95% CI -0.08 - 0.10, P=0.87, I²=22%). The pooled analysis did not find a significant difference between LMWF-5A and placebo regarding the incidence of joint swelling (P=0.84), joint stiffness (P=0.53), arthralgia (P=0.53), extremity pain (P=0.45), NSAEs (P=0.21), SAEs (P=0.92), or mortality (P=1.00). However, the subgroup analysis showed a significant reduction of 42% in NSAEs upon administration of 10 mL of LMWF-5A (risk ratio (RR)=0.58, 95% CI 0.35-0.97, P=0.04).

In summary, our meta-analysis did not find significant differences between LMWF-5A and placebo regarding the incidence of NSAEs, SAEs, or mortality. On the other hand, LMWF-5A did not demonstrate superiority over saline in terms of efficacy. Therefore, it is not an effective drug for managing knee osteoarthritis.

## Introduction and background

Osteoarthritis (OA) is a chronic degenerative disorder that primarily affects weight-bearing joints, such as the knee, hip, and vertebral joints. The worldwide prevalence of knee OA is estimated to be 22.9% among adults aged ≥ 40 years [1]. The knee joints are the most affected and carry four-fifths of the disease burden [1]. Knee OA is a degenerative disease that eventually results in the destruction of the joint complex [2], which is secondary to joint trauma, being overweight, or age-related loss of cartilage. The mechanism of cartilage loss involves inflammatory cytokines such as interleukin-1 (IL-1), IL-6, tumor necrosis factor (TNF)-α, and adipokines that trigger inflammation and induce degradation of the cartilage matrix [2]. Determining the severity of knee osteoarthritis involves a comprehensive evaluation that considers various factors depending on the patient’s symptoms and radiographic changes [3]. The Kellgren-Lawrence (KL) grading system is a widely used tool for assessing the degree of joint space narrowing, osteophyte formation, and other changes seen on X-rays. KL is classified into grades ranging from 0-4, in which KL grade 4 represents severe knee OA [4].

In the early stages of the disease, knee OA is managed conservatively. The aim of the conservative approach is to control patients’ symptoms and improve their quality of life; however, no medication has been proven to reverse joint destruction [5]. The recommended first-line treatment for mild knee OA includes a combination of physiotherapy and analgesics such as nonsteroidal anti-inflammatory drugs (NSAIDs) and acetaminophen [6-8]. Chronic use of NSAIDs can lead to serious adverse effects such as cardiotoxicity, gastrointestinal bleeding, and renal failure [9]. Acetaminophen, on the other hand, has fewer adverse events but is inferior to NSAIDs in terms of effectiveness [10]. Additionally, intra-articular corticosteroids are indicated for managing moderate knee OA with fewer systemic side effects [6]. They also have strong evidence of short-term pain improvement for up to six weeks [11]. Nonetheless, high doses of intra-articular corticosteroids can further increase cartilage destruction, thereby accelerating disease progression [12]. Hyaluronic acid (HA) is an alternative intra-articular injection that has greater long-term benefits than corticosteroids, lasting up to eight weeks or more [13]. Opioids, such as tramadol, are indicated in select patients with moderate to severe knee OA who do not respond to other medications [14]. They are only recommended for short-term use because there is a significant risk of developing addiction, dependence, respiratory depression, and cognitive impairment [15]. Regarding severe knee OA, previous interventions tend to be less effective in pain control [16]. Total knee replacement (TKR) is a surgical procedure that involves the removal of diseased knee joint surfaces, which are then replaced with artificial components composed of metal, plastic, or ceramic materials. TKR is the last treatment modality used when conservative options are unsuccessful [7].

Human serum albumin (HSA) has been indicated for the treatment of several conditions, including shock, blood volume resuscitation, and hypoalbuminemia, in the past three decades with optimal safety and efficacy profiles in humans [17-18]. Recently, several trials have suggested the use of HSA in the form of a low molecular weight fraction of 5% HSA (LMWF-5A) as an intra-articular injection for the treatment of knee OA because of its anti-inflammatory properties in reducing pain and swelling and improving function [19]. Aspartate-alanine diketopiperazine (DA-DKP) is a component found in LMWF-5A that plays a role in reducing cytokines responsible for triggering inflammation, mainly TNF-α. As a result, it leads to a significant decrease in inflammation [19]. In addition, LMWF-5A decreases pain by promoting the release of prostaglandins that inhibit nociceptors and may play a role in reducing inflammation [19]. In this article, a systematic review and meta-analysis were performed to evaluate the efficacy and safety of LMWF-5A intra-articular injections for the management of knee osteoarthritis with different regimens listed as subgroups against placebo.

## Review

Materials and methods

Protocol

This systematic review was performed according to a prespecified protocol registered at The International Prospective Register of Systematic Reviews (PROSPERO) (CRD42022364088). This review was conducted in accordance with the Preferred Reporting Items for Systematic Reviews and Meta-Analysis (PRISMA) checklist.

Search Strategy

The Cochrane Central Register of Controlled Trials (CENTRAL), EBSCO, Embase, Medical Literature Analysis and Retrieval System Online (MEDLINE), and ClinicalTrials.gov registry (CT.gov) databases were searched on December 21, 2022, without any restrictions on language or date. The search strategy is described in Appendices A-D.

Inclusion and Exclusion Criteria

Only randomized controlled trials (RCTs) comparing intra-articular injections of LMWF-5A and placebo were considered eligible. The population included adult patients of both sexes between the ages of 35 and 85 with moderate to moderately severe OA. Trials that fulfilled the desired outcomes, such as the Western Ontario and McMaster Universities Arthritis Index (WOMAC) A and WOMAC C, in assessing the efficacy, serious adverse events (SAEs), non-serious adverse events (NSAEs), and mortality to evaluate the safety of the drug, were included. Non-RCTs were excluded.

Outcomes

Pain and function were evaluated using the WOMAC to determine the efficacy of the drug. The outcomes were the mean change in both WOMAC A (pain) and WOMAC C (function) scores from baseline to 12 weeks. The incidence of joint swelling, joint stiffness, arthralgia, pain in the extremities, NSAEs, SAEs, and mortality indicated the safety of the drug. Safety measures were recorded from the initiation of each trial until the final follow-up.

Data Extraction

Title and abstract screening, full-text assessment, and data extraction of RCTs were independently performed by pairs of reviewers. Conflicts were resolved by a third reviewer.

Risk of Bias Assessment

**Figure 1:**
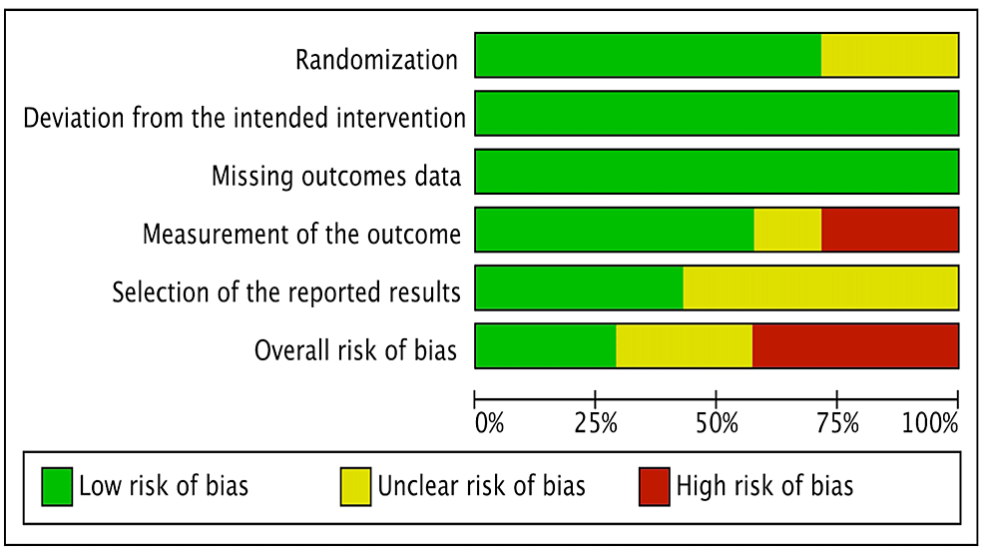
Risk of bias graph

**Figure 2:**
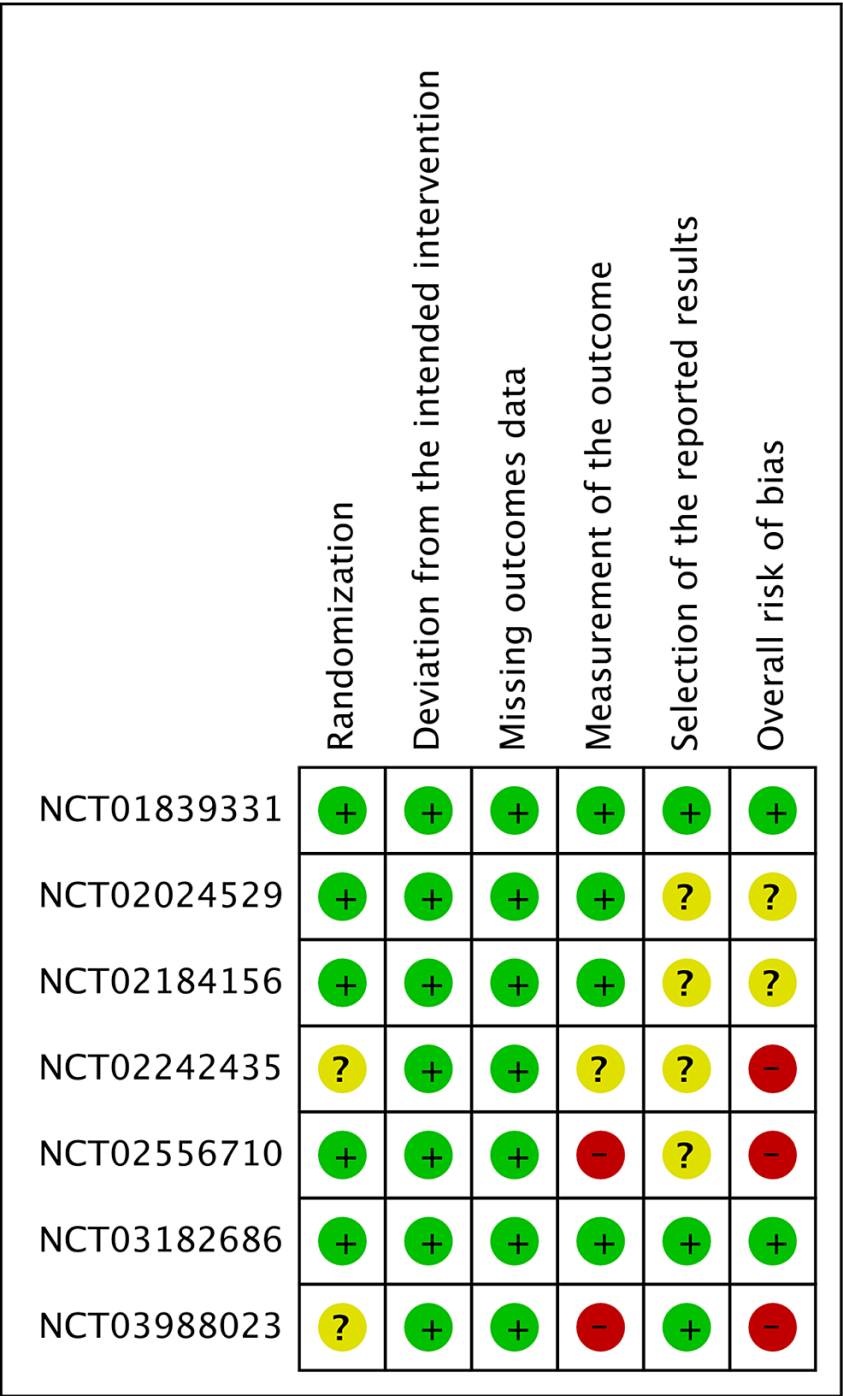
Risk of bias summary Study or subgroup: NCT01839331 [17]; NCT03988023 [21]; NCT02556710 [22]; NCT02242435 [23]; NCT03182686 [24]; NCT02184156 [25]; NCT02024529 [26]

The revised Cochrane risk of bias tool was utilized by two reviewers to independently assess the risk of bias of the included RCTs [20]. Three studies had a high risk of bias, two had some concerns, and the remaining two had a low risk of bias. Conflicts between the reviewers were resolved through discussion until they reached an agreement. The risk of bias assessment is shown in Figures 1-2.

Statistical Analysis

Review Manager (RevMan) Version 5.4 (Cochrane Collaboration, 2020) was used to perform data analysis. A random-effects model (RE) was used, and a 95% confidence level and P < 0.05 as a borderline were considered for statistical significance. Statistical heterogeneity was assessed using the I2 index. Mean changes in WOMAC A and WOMAC C were used as continuous variables to assess the efficacy and were represented by the standardized mean difference (SMD). Dichotomous outcomes (SAEs, NSAEs, and mortality) were represented as risk ratios (RRs), and the pooled estimate was calculated using the inverse variance weighting method. The intervention arm was sub-grouped into a single injection regimen, which was further divided into 4 ml and 10 ml doses, and a multiple injection regimen with a 4 ml dose. The quality of evidence for each outcome was assessed using the Grading of Recommendations Assessment, Development, and Evaluation (GRADE) criteria.

Results

Study Selection 

**Figure 3:**
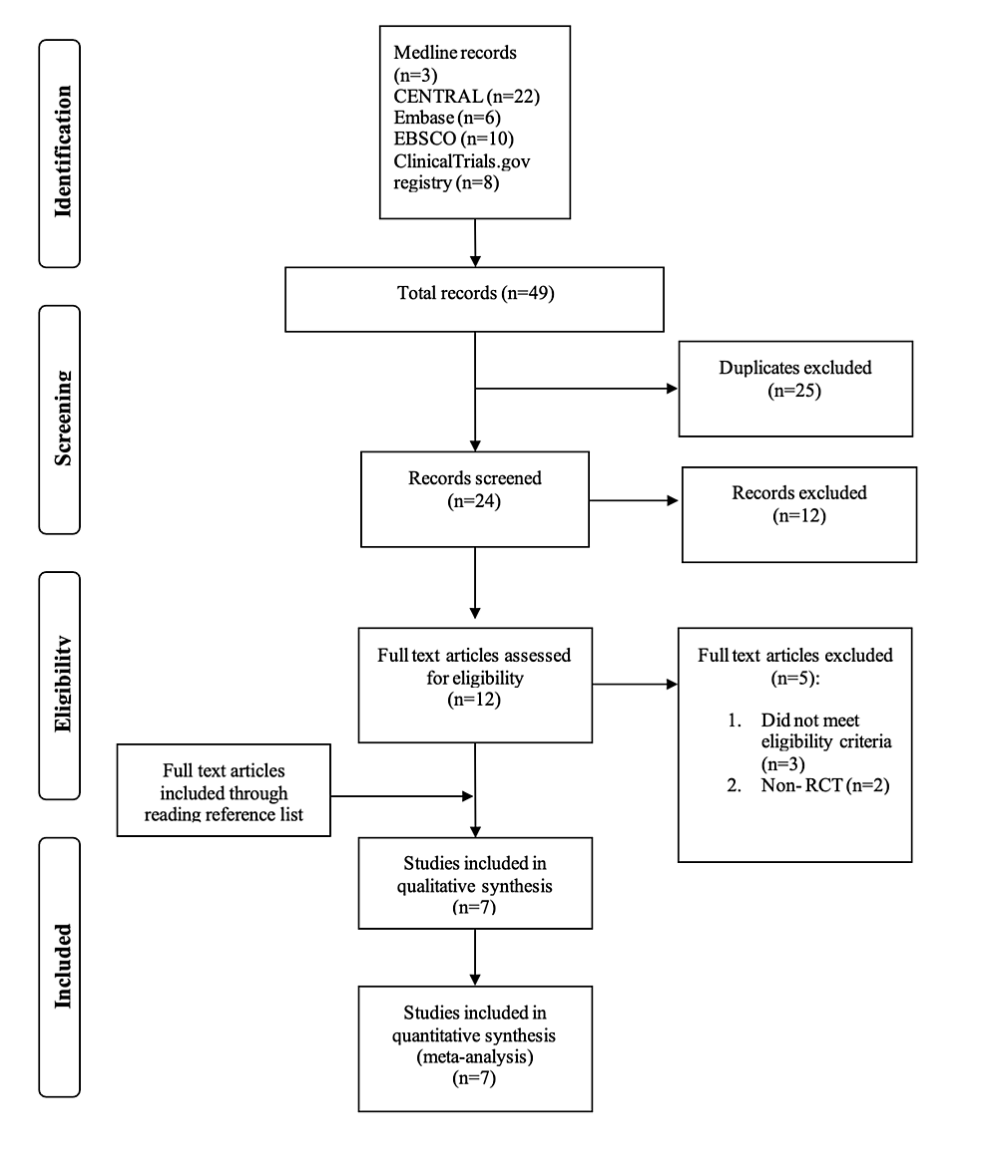
A study flow diagram of the process of selecting the studies in this meta-analysis This was done according to Preferred Reporting Items for Systematic Reviews and Meta-Analyses (PRISMA) guidelines.

Upon a systematic search, 49 studies were identified, including 25 duplicates, resulting in 24 studies. Of the 24 RCTs, 17 were excluded because of unmatched eligibility. Seven RCTs that matched the inclusion criteria were included in our study. The flow chart of the process of selecting of the studies according to PRISMA guidelines is demonstrated in Figure 3.

Trial Characteristics

In total, 2,939 participants were included in the trials. The mean age of the patients ranged from 60.6 to 64.7 years. Approximately 1,153 of these participants (39.1%) were male. A total of 2,817 of the participants were not Hispanic or Latino. The baseline characteristics of the included participants are available in Table 1.

**Table 1:** Baseline characteristics of the trials This table shows the demographics and baseline characteristics of participants in all the included trials, including the number of participants, gender, ethnicity, mean age, BMI, WOMAC A, and WOMAC C. WOMAC: Western Ontario and McMaster Universities Osteoarthritis Index; BMI: body mass index: LMWF-5A: low molecular weight fraction of 5%

Trial registry	Dose (mg/mL)	Number of participants (started)		Number of participants (completed)		Gender		Ethnicity			Mean age	BMI	WOMAC pain mean (LMWF-5A)	WOMAC pain mean (placebo)	WOMAC function mean (LMWF-5A)	WOMAC function mean (placebo)
		LMWF-5A	Placebo	LMWF-5A	Placebo	Male	Female	Hispanic or Latino	Not Hispanic or Latino	Unknown or not reported						
NCT01839331 [17]	4	83	83	79	77	53	113	4	325	0	62.48 (9.02)	33.16 (7.27)	2.22	2.32	2.13	2.25
	10	82	81	77	76	67	96						2.19	2.23	2.23	2.22
NCT03988023 [21]	4	520	523	235	235	397	647	23	319	0	63.2 (9.3)	34.6 (7.8)	2.51	2.57	2.51	2.59
NCT02556710 [22]	4	237	243	226	237	169	311	32	447	1	61.2 (8.95)	33.43 (7.30)	2.37	2.33	2.37	2.34
NCT02242435 [23]	4	172	170	152	154	144	198	51	986	6	64.7 (9.2)	34.3 (8.3)	2.34	2.40	2.43	2.45
NCT03182686 [24]	4	144	24	137	24	80	88	0	168	0	62.92 (9.22)	n/a	2.5	2.4	2.6	2.5
NCT02184156 [25]	4	20	19	20	19	15	25	3	37	0	62.55 (9.85)	29.85 (5)	2.23	2.17	2.17	2.3
NCT02024529 [26]	4	269	269	256	244	228	310	0	535	3	60.6 (9.2)	34.2 (7.9)	2.38	2.38	2.45	2.41

Efficacy

WOMAC A

**Figure 4:**
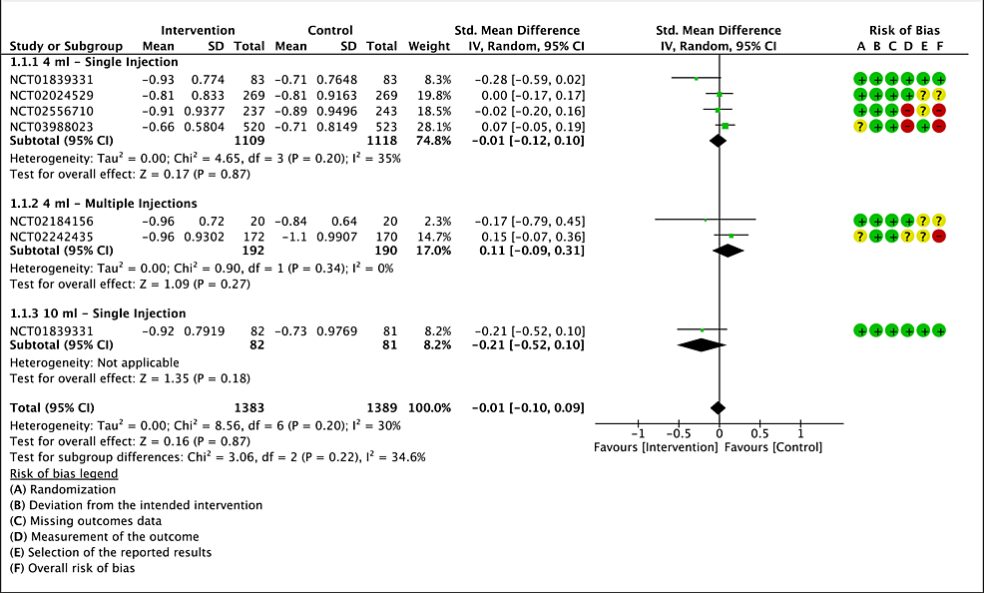
A forest plot of the mean change of WOMAC A at 12 weeks CI: confidence interval; IV: inverse variance; SD: standard deviation Study or subgroup: NCT01839331 [17]; NCT03988023 [21]; NCT02556710 [22]; NCT02242435 [23]; NCT02184156 [25]; NCT02024529 [26]

The pain was assessed using the WOMAC A score. Six of the included RCTs had a mean change in WOMAC A scores from baseline to week 12 as the primary outcome. A meta-analysis showed that LMWF-5A did not significantly improve pain (P=0.87). Subgroup analysis did not indicate a significant decrease in pain when LMWF-5A was used as a single injection either at a dose of 4 ml (P=0.87) or 10 ml (P=0.18). For multiple injections of LMWF-5A, there was no significant reduction in WOMAC A mean change compared to the placebo (P=0.27). The results of the analysis are presented in Figure 4.

The grade of certainty of evidence was rated as low for WOMAC A (Appendix E).

WOMAC C

**Figure 5:**
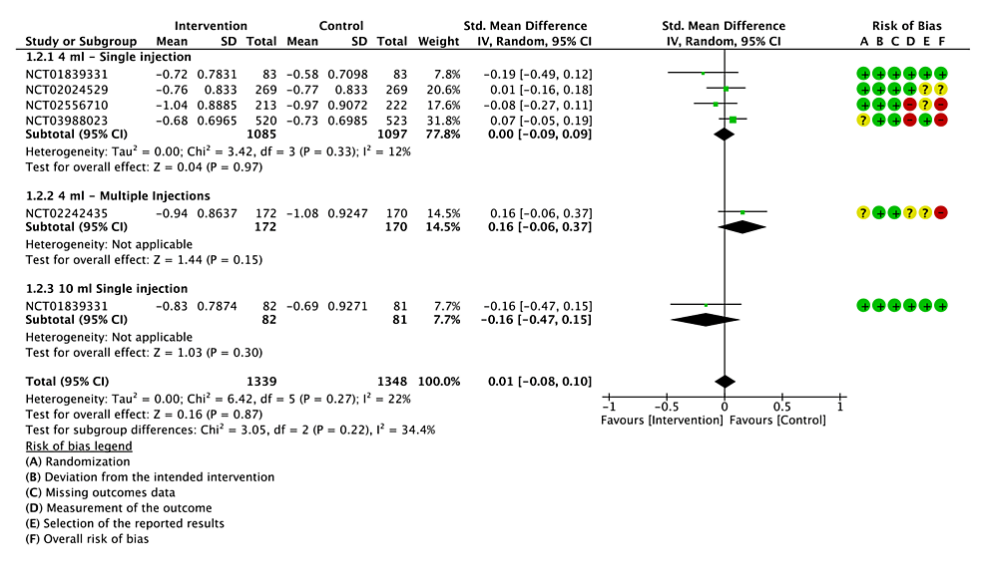
A forest plot of the mean change of WOMAC C at 12 weeks CI: confidence interval; IV: inverse variance; SD: standard deviation Study or subgroup: NCT01839331 [17]; NCT03988023 [21]; NCT02556710 [22]; NCT02242435 [23]; NCT02024529 [26]

The mean change in WOMAC C score from baseline to 12 weeks was used as the endpoint to show improvement in knee function. Our analysis showed that LMWF-5A did not significantly enhance knee function compared to the control (P=0.87). The subgroup analysis also showed no difference when the drug was used as a single injection at a dose of 4 ml (P=0.97) or 10 ml (P=0.30). Furthermore, when LMWF-5A was administered as multiple injections, it did not significantly enhance knee function (P=0.30). The results of the analysis are shown in Figure 5.

The GRADE criteria were judged moderate for WOMAC C (Appendix E).

Safety

Joint Swelling 

**Figure 6:**
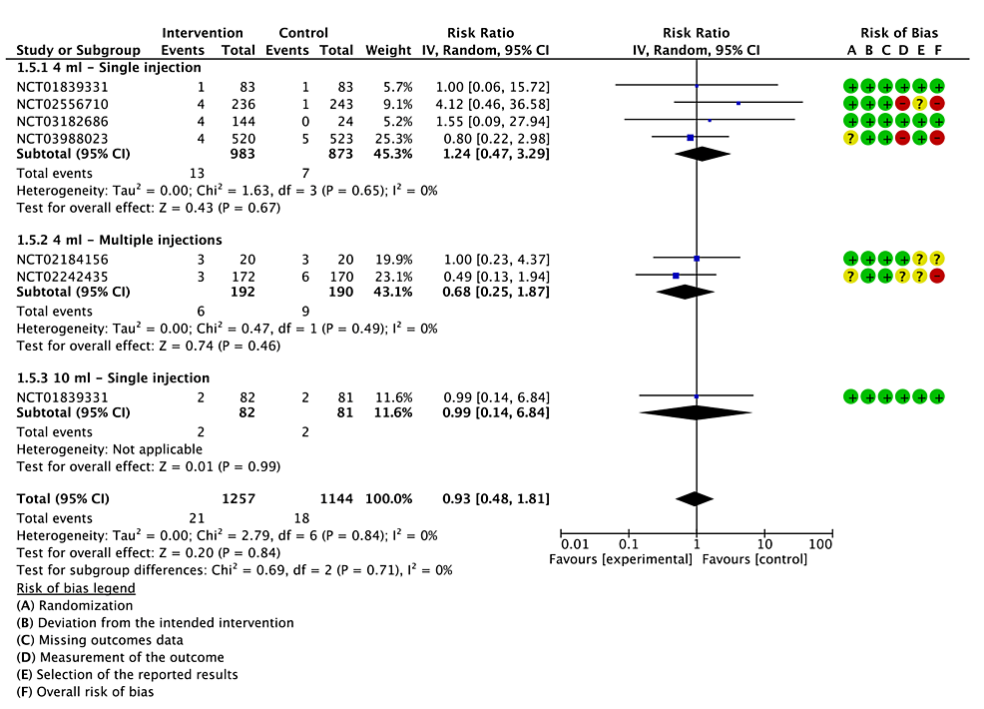
A forest plot of the analysis of joint swelling CI: confidence interval; IV: inverse variance; SD: standard deviation Study or subgroup: NCT01839331 [17]; NCT03988023 [21]; NCT02556710 [22]; NCT02242435 [23]; NCT03182686 [24]; NCT02184156 [25]

Six studies reported on the incidence of joint swelling after the administration of LMWF-5A; there was no significant reduction in joint swelling incidence (P=0.84). Similarly, subgroup analysis did not find significant differences in the incidence of joint swelling when LMWF-5A was administered as a single injection at a dose of 4 ml (P=0.67) or 10 ml (P=0.99). Moreover, multiple injections of LMWF-5A did not significantly decrease swelling (P=0.46). The results of the analysis are demonstrated in Figure 6. 

The GRADE criteria were evaluated as low for joint swelling (Appendix E).

Joint Stiffness

**Figure 7:**
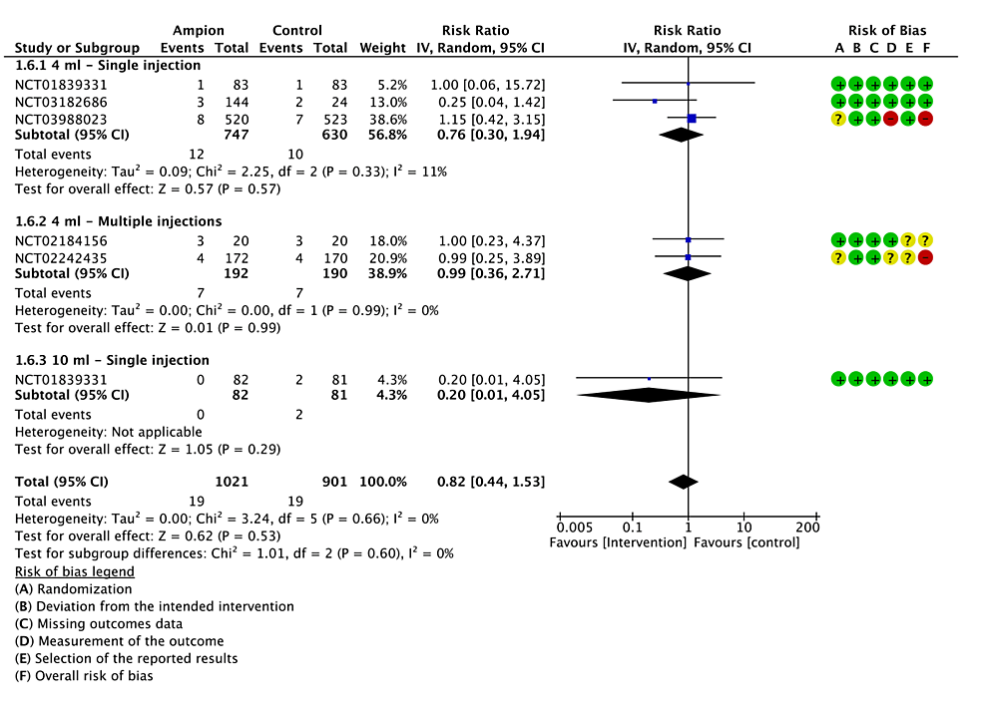
A forest plot of the analysis of joint stiffness CI: confidence interval; IV: inverse variance; SD: standard deviation Study or subgroup: NCT01839331 [17]; NCT03988023 [21]; NCT02242435 [23]; NCT03182686 [24]; NCT02184156 [25]

Five studies reported the rate of joint stiffness. Our meta-analysis showed no significant difference between LMWF-5A and placebo regarding the incidence of joint stiffness (P=0.53). Additionally, subgroup analysis did not show a significant reduction in the rate of joint stiffness with either single injections of 4 ml (P=0.57) or 10 ml (P=0.29), or multiple injections (P=0.99). The results of the analysis are presented in Figure 7.

The GRADE criteria were assessed as moderate for joint stiffness (Appendix E).

Arthralgia

**Figure 8:**
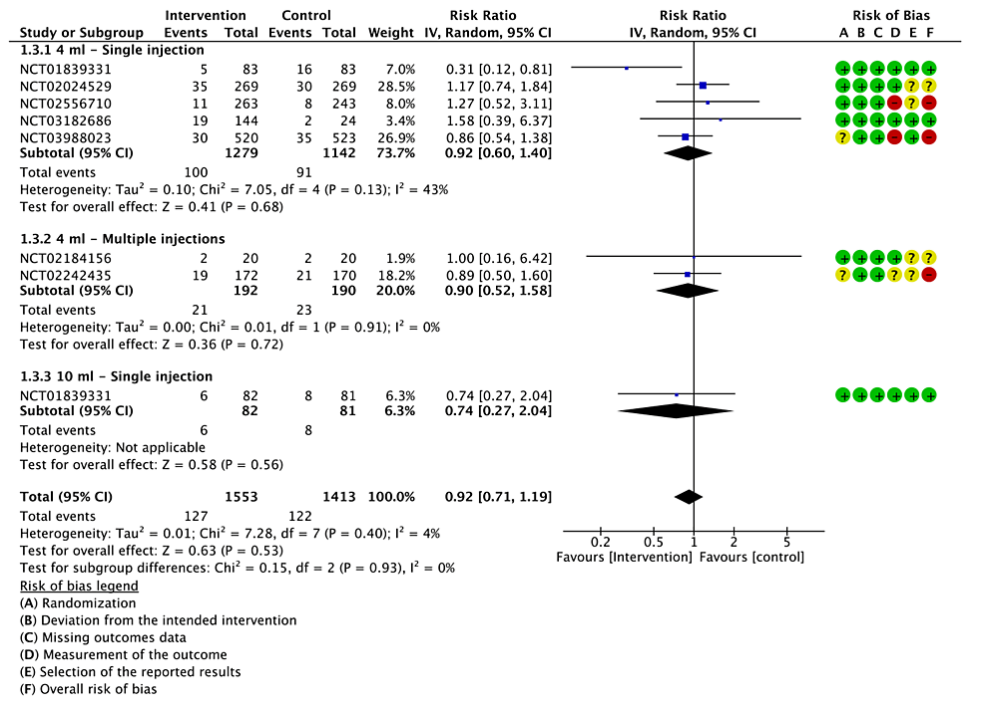
A forest plot of the analysis of arthralgia CI: confidence interval; IV: inverse variance; SD: standard deviation Study or subgroup: NCT01839331 [17]; NCT03988023 [21]; NCT02556710 [22]; NCT02242435 [23]; NCT03182686 [24]; NCT02184156 [25]; NCT02024529 [26]

Seven studies reported the incidence of arthralgia after LMWF-5A administration. Arthralgia was the most reported adverse event (AE), which was slightly higher in LMWF-5A (127 events), yet there was no significant difference when compared to saline (122 events) (P=0.53). Regarding subgroup analysis, LMWF-5A did not show a significant difference compared to placebo with either 4 ml (P=0.68) or 10 ml single injections (P=0.56). The results of the analysis are presented in Figure 8.

The GRADE criteria evaluation showed a low certainty of evidence for arthralgia (Appendix E).

Extremity Pain

**Figure 9:**
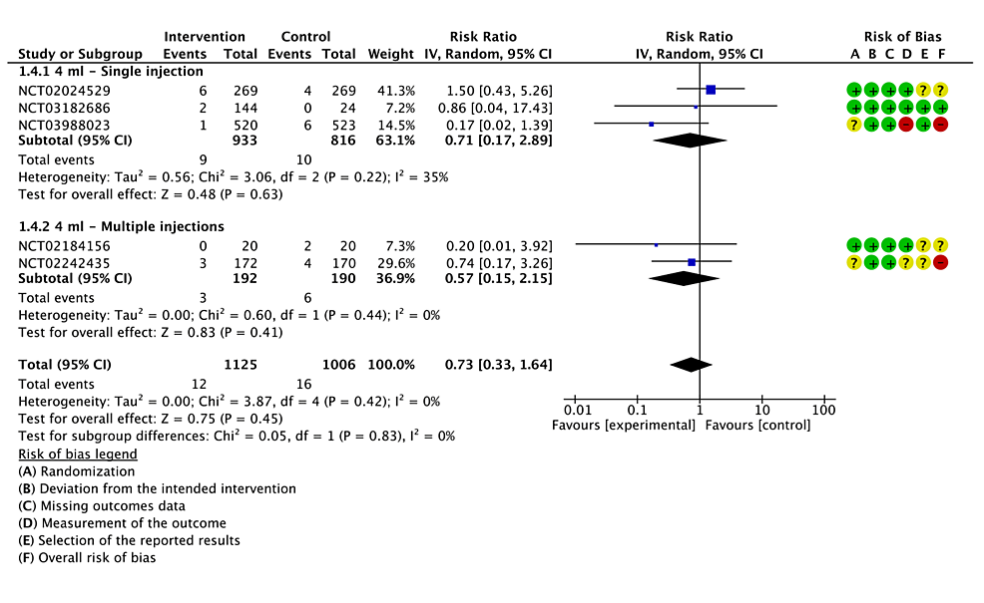
A forest plot of the analysis of extremity pain CI: confidence interval; IV: inverse variance; SD: standard deviation Study or subgroup: NCT03988023 [21]; NCT02242435 [23]; NCT03182686 [24]; NCT02184156 [25]; NCT02024529 [26]

Five trials reported the incidence of extremity pain. LMWF-5A did not significantly decrease extremity pain compared to saline (P=0.45). On subgroup analysis, neither single injections (P=0.63) nor multiple injections (P=0.41) significantly reduced pain in the extremities. The results of the analysis are demonstrated in Figure 9. 

The GRADE criteria showed moderate certainty of evidence for extremity pain (Appendix E).

Non-serious Adverse Events (NSAEs)

**Figure 10:**
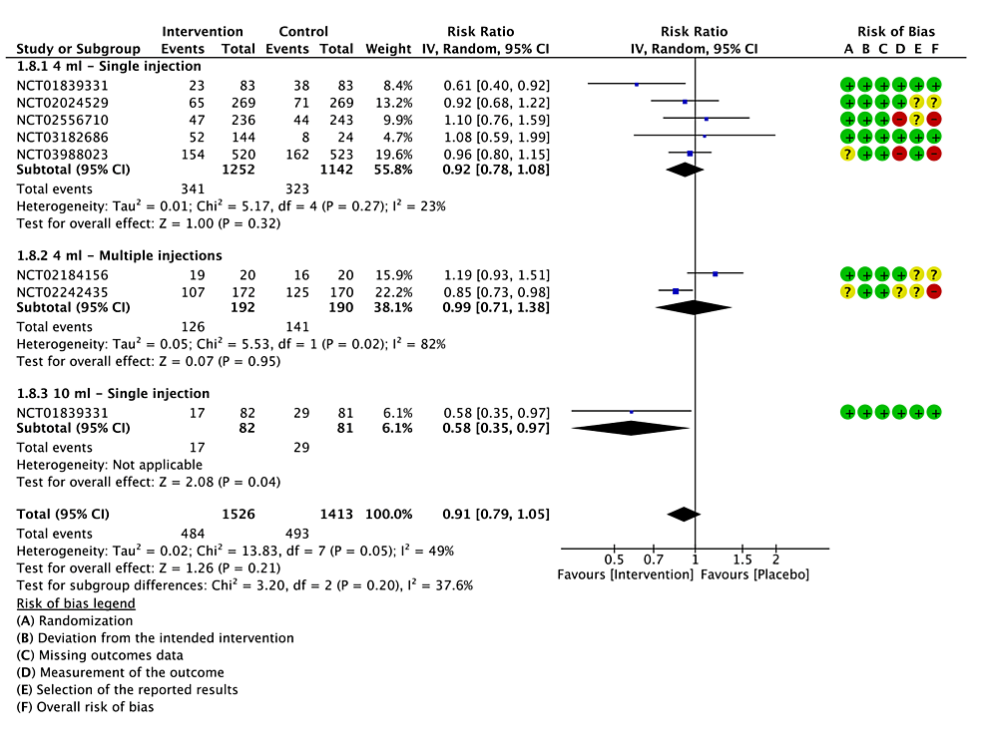
A forest plot of the analysis of NSAEs CI: confidence interval; IV: inverse variance; SD: standard deviation Study or subgroup: NCT01839331 [17]; NCT03988023 [21]; NCT02556710 [22]; NCT02242435 [23]; NCT03182686 [24]; NCT02184156 [25]; NCT02024529 [26]

All the included studies reported NSAEs from the initiation of each study until the last follow-up. NSAEs were defined as any event that did not result in death, a life-threatening condition, hospital admission, or prolongation of current hospitalization, did not impact the normal level of function, or caused birth defects. with 484 events occurring in the LMWF-5A group and 493 events in the control group. LMWF-5A did not significantly impact the reduction in NSAEs (P=0.21), and subgroup analysis did not find a significant difference upon administration of 4 ml as a single injection (P=0.32). However, when administered at a dose of 10 ml, it significantly decreased the rate of NSAEs (P=0.04). In contrast, multiple injections showed no significant difference from the control group (P=0.95). The results of the analysis are shown in Figure 10.

The GRADE approach for NSAEs was rated as low (Appendix E).

Serious Adverse Events (SAEs)

**Figure 11:**
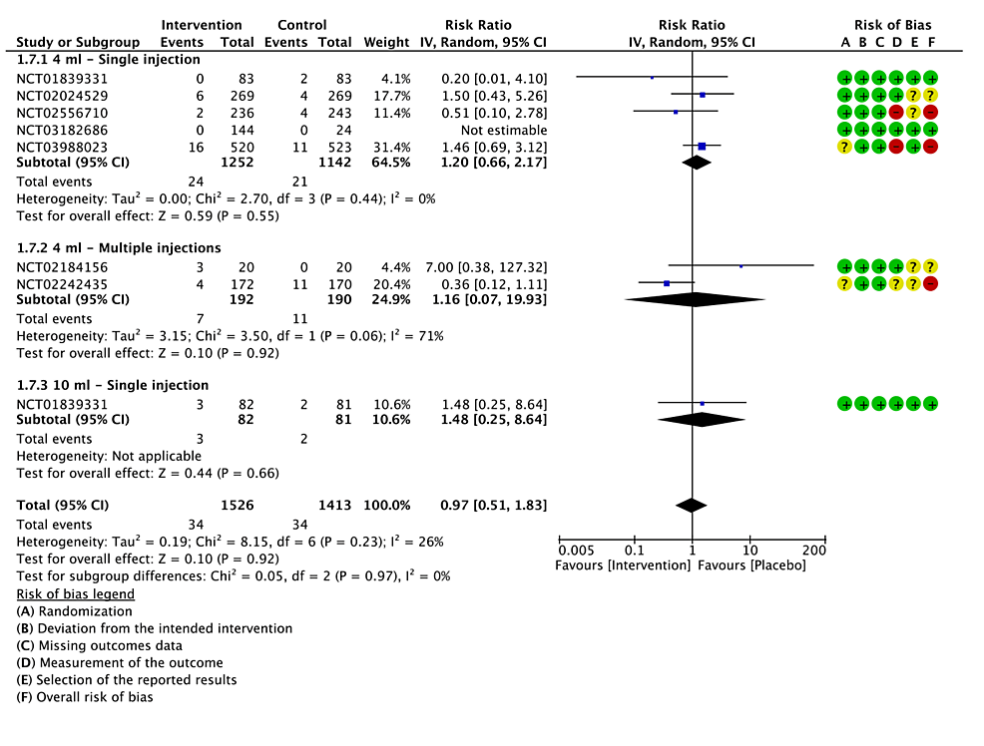
A forest plot of the analysis of SAEs CI: confidence interval; IV: inverse variance; SD: standard deviation Study or subgroup: NCT01839331 [17]; NCT03988023 [21]; NCT02556710 [22]; NCT02242435 [23]; NCT03182686 [24]; NCT02184156 [25]; NCT02024529 [26]

All seven included studies evaluated SAEs from the initiation of the trial until the last follow-up. SAEs are defined as any adverse event that is considered serious if it results in death, a life-threatening event that requires hospitalization or extends a current hospitalization, a significant disruption of normal functioning, an event that leads to the requirement of surgical or medical intervention, or a congenital anomaly in the offspring of a study participant. The total number of SAEs in the LMWF-5A arm was 34 out of 1,526 participants in the LMWF-5A arm and 34 out of 1,413 participants in the placebo arm. Pooled analysis showed that the rate of SAEs was not significantly reduced by LMWF-5A treatment (P=0.92). Similarly, subgroup analysis did not find a significant decrease with a single injection of 4 ml (P=0.55) or 10 ml (P=0.66). Multiple injections did not result in significant changes (P=0.91). The results of the analysis are presented in Figure 11.

Upon assessment of the GRADE criteria for certainty of evidence, the SAEs were rated as low (Appendix E).

Mortality

**Figure 12:**
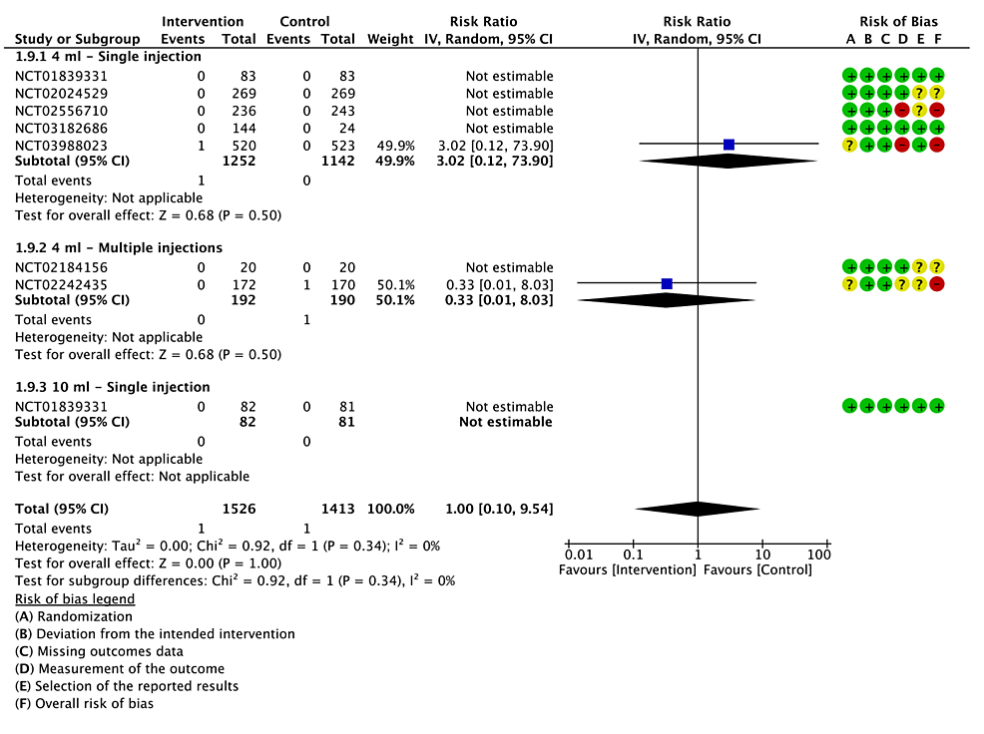
A forest plot of the analysis of mortality CI: confidence interval; IV: inverse variance; SD: standard deviation Study or subgroup: NCT01839331 [17]; NCT03988023 [21]; NCT02556710 [22]; NCT02242435 [23]; NCT03182686 [24]; NCT02184156 [25]; NCT02024529 [26]

Only two deaths occurred in two different studies (P=1.00), which was insignificant as shown in Figure 12. 

The GRADE certainty of evidence was evaluated as low for mortality (Appendix E).

Discussion

Our systematic review and meta-analysis evaluated the efficacy and safety of LMWF-5A in the management of moderate to moderately severe knee osteoarthritis. However, LMWF-5A did not significantly improve pain or function. It also showed insignificant differences when compared to placebo in the safety profile. LMWF-5A is a novel drug that has recently been proposed by several RCTs for the management of knee OA as an intra-articular injection. An integrated analysis that evaluated the efficacy and safety of a single injection of LMWF-5A for the management of moderate to moderately severe knee OA showed a significant reduction in pain and improvement in function [27]. Another study found that the incidence of total knee replacement following LMWF-5A was significantly lower in patients with severe osteoarthritis. However, an insignificant difference was noted between moderate osteoarthritis and saline [28]. The integrated analysis evaluated efficacy by assessing the mean change in WOMAC A and WOMAC C from baseline to 12 weeks only when LMWF-5A was administered as a single injection; it included three trials and found a significant reduction in pain [27]. In contrast, our meta-analysis included four RCTs that administered the drug as a single injection and did not find a significant improvement in pain when compared to placebo [27]. The previous study also found that LMWF-5A significantly improved function, but our study demonstrated no significant differences compared to the placebo regarding the WOMAC C score. Although patient global assessment (PGA) and WOMAC B scores were evaluated using integrated analysis [27], we did not include them because the data were only available in one trial, whereas the data were unpublished for the remaining RCTs. We evaluated the safety of the drug by assessing the rate of NSAEs, SAEs, and mortality among the seven included studies. Arthralgia was the most reported NSAE in all the included RCTs, which was slightly higher in the LMWF-5A group. Our findings did not show a noticeable decrease in the incidence of SAEs, NSAEs, or mortality upon the administration of LMWF-5A, similar to the findings of an earlier integrated analysis [27]. However, our meta-analysis found that the administration of 10 ml of LMWF-5A as a single injection resulted in fewer NSAEs. Only two deaths were reported in all studies, one in the LMWF-5A arm and the other in the placebo arm, which were unrelated to the administration of the drug and did not negatively impact its safety.

The GRADE criteria, which include five major domains-the risk of bias, imprecision, inconsistency, indirectness, and publication bias-were implemented to assess the certainty of evidence of the included studies. The utilization of the GRADE criteria for outcome appraisal facilitates a lucid assessment of the degree of certainty pertaining to the evidence and a precise evaluation of the study outcomes, consequently resulting in precise and pragmatic recommendations from the authors. It is noteworthy that while the GRADE approach offers a reliable method for determining the quality of evidence, it does not preclude the indispensability of clinical judgment. Intra-articular LMWF-5A is a new treatment for knee osteoarthritis that has not yet been extensively studied. More RCTs are required to evaluate its efficacy and safety for use in the management of knee osteoarthritis.

This study is the first systematic review and meta-analysis to assess the use of LMWF-5A for managing knee OA and to perform a subgroup analysis of two different doses (4 ml and 10 ml). Two of the included RCTs had a low risk of bias. Two studies had some concerns, while three studies were scored as high-risk using the Cochrane risk of bias assessment tool. Because of the low number of included RCTs, funnel plots were not used in this study, which made the detection of publication bias infeasible. No significant heterogeneity was observed among the included studies.

Limitations

This study had some limitations. One limitation was the low number of RCTs because of the recent development of the drug. Another limitation was the unpublished data of some of the included RCTs, which hindered the accessibility of particular outcome results at different time points.

## Conclusions

Our findings show that LMWF-5A is not an effective intervention option for knee osteoarthritis and does not show any superiority over placebo in terms of efficacy or safety. Further extensive RCTs involving various outcomes are recommended in the future. Future studies on LMWF-5A should focus on comparing the drug to current intra-articular injections, such as corticosteroids, to assess the safety and efficacy of the drug and to ascertain whether LMWF-5A can be applied as an intra-articular injection for knee osteoarthritis.

## Appendices

Appendix A 

**Table 2:** MEDLINE search strategy

Search number	Query	Sort by	Filters	Search details	Results	Time
4	#1 AND #2 AND #3	Most Recent		("osteoarthritis, knee"[MeSH Terms] OR "Osteoarthritis"[MeSH Terms] OR "Osteoarthritis"[Title/Abstract] OR "Osteoarthritis"[Title/Abstract] OR "osteoarthritis of the knee"[Title/Abstract] OR ("Osteoarthritis"[MeSH Terms] OR "Osteoarthritis"[All Fields] OR "osteoarthritides"[All Fields]) OR ("osteoarthritis, knee"[MeSH Terms] OR ("Osteoarthritis"[All Fields] AND "knee"[All Fields]) OR "knee osteoarthritis"[All Fields] OR ("Osteoarthritis"[All Fields] AND "knee"[All Fields]) OR "osteoarthritis of the knee"[All Fields]) OR ("osteoarthritis, knee"[MeSH Terms] OR ("Osteoarthritis"[All Fields] AND "knee"[All Fields]) OR "knee osteoarthritis"[All Fields] OR ("knee"[All Fields] AND "Osteoarthritis"[All Fields]))) AND ("serum albumin"[MeSH Terms] OR "serum albumin"[Title/Abstract] OR "human serum albumin"[Title/Abstract] OR (("Low"[All Fields] AND ("molecular weight"[MeSH Terms] OR ("molecular"[All Fields] AND "weight"[All Fields]) OR "molecular weight"[All Fields])) AND "serum albumin"[Title/Abstract]) OR "low molecular weight albumin"[Title/Abstract] OR "LMWF-5A"[Title/Abstract] OR "Ampion"[Title/Abstract] OR (("Low"[All Fields] AND ("molecular weight"[MeSH Terms] OR ("molecular"[All Fields] AND "weight"[All Fields]) OR "molecular weight"[All Fields]) AND ("dose fractionation, radiation"[MeSH Terms] OR ("dose"[All Fields] AND "fractionation"[All Fields] AND "radiation"[All Fields]) OR "radiation dose fractionation"[All Fields] OR "fractionation"[All Fields] OR "chemical fractionation"[MeSH Terms] OR ("chemical"[All Fields] AND "fractionation"[All Fields]) OR "chemical fractionation"[All Fields] OR "fraction"[All Fields] OR "fraction s"[All Fields] OR "fractionate"[All Fields] OR "fractionated"[All Fields] OR "fractionates"[All Fields] OR "fractionating"[All Fields] OR "fractionationed"[All Fields] OR "fractionations"[All Fields] OR "fractionator"[All Fields] OR "fractionators"[All Fields] OR "fractioned"[All Fields] OR "fractioning"[All Fields] OR "fractionized"[All Fields] OR "fractions"[All Fields])) AND "of human serum albumin"[Title/Abstract])) AND ("randomized controlled trials as topic"[MeSH Terms] OR "randomized controlled trials as topic"[MeSH Terms] OR "randomized controlled trials as topic"[MeSH Terms] OR "randomized controlled trials as topic"[MeSH Terms] OR "clinical trials as topic"[MeSH Terms] OR "clinical trials as topic"[MeSH Terms] OR "clinical trials as topic"[MeSH Terms] OR "clinical trials as topic"[MeSH Terms])	3	17:27:14
3	"randomized controlled trials as topic"[MeSH Terms] OR "randomized controlled trials as topic"[MeSH Terms] OR "randomized controlled trials as topic"[MeSH Terms] OR "randomized controlled trials as topic"[MeSH Terms] OR "clinical trials as topic"[MeSH Terms] OR "clinical trials as topic"[MeSH Terms] OR "clinical trials as topic"[MeSH Terms] OR "clinical trials as topic"[MeSH Terms]	Most Recent		"randomized controlled trials as topic"[MeSH Terms] OR "randomized controlled trials as topic"[MeSH Terms] OR "randomized controlled trials as topic"[MeSH Terms] OR "randomized controlled trials as topic"[MeSH Terms] OR "clinical trials as topic"[MeSH Terms] OR "clinical trials as topic"[MeSH Terms] OR "clinical trials as topic"[MeSH Terms] OR "clinical trials as topic"[MeSH Terms]	378,995	17:26:58
2	(((((((serum albumin[MeSH Terms]) OR (Serum albumin[Title/Abstract])) OR (human serum albumin[Title/Abstract])) OR (low molecular weight serum albumin[Title/Abstract])) OR (low molecular weight albumin[Title/Abstract])) OR (LMWF-5A[Title/Abstract])) OR (Ampion[Title/Abstract])) OR (Low molecular weight fraction of human serum albumin[Title/Abstract])	Most Recent		"serum albumin"[MeSH Terms] OR "serum albumin"[Title/Abstract] OR "human serum albumin"[Title/Abstract] OR (("Low"[All Fields] AND ("molecular weight"[MeSH Terms] OR ("molecular"[All Fields] AND "weight"[All Fields]) OR "molecular weight"[All Fields])) AND "serum albumin"[Title/Abstract]) OR "low molecular weight albumin"[Title/Abstract] OR "LMWF-5A"[Title/Abstract] OR "Ampion"[Title/Abstract] OR (("Low"[All Fields] AND ("molecular weight"[MeSH Terms] OR ("molecular"[All Fields] AND "weight"[All Fields]) OR "molecular weight"[All Fields]) AND ("dose fractionation, radiation"[MeSH Terms] OR ("dose"[All Fields] AND "fractionation"[All Fields] AND "radiation"[All Fields]) OR "radiation dose fractionation"[All Fields] OR "fractionation"[All Fields] OR "chemical fractionation"[MeSH Terms] OR ("chemical"[All Fields] AND "fractionation"[All Fields]) OR "chemical fractionation"[All Fields] OR "fraction"[All Fields] OR "fraction s"[All Fields] OR "fractionate"[All Fields] OR "fractionated"[All Fields] OR "fractionates"[All Fields] OR "fractionating"[All Fields] OR "fractionationed"[All Fields] OR "fractionations"[All Fields] OR "fractionator"[All Fields] OR "fractionators"[All Fields] OR "fractioned"[All Fields] OR "fractioning"[All Fields] OR "fractionized"[All Fields] OR "fractions"[All Fields])) AND "of human serum albumin"[Title/Abstract])	123,737	17:23:22
1	(((((((knee osteoarthritis[MeSH Terms]) OR (osteoarthritis[MeSH Terms])) OR (osteoarthritis[Title/Abstract])) OR (osteoarthritis[Title/Abstract])) OR (Osteoarthritis of the knee[Title/Abstract])) OR (Osteoarthritis)) OR (osteoarthritis of the knee)) OR (knee osteoarthritis)	Most Recent		"osteoarthritis, knee"[MeSH Terms] OR "Osteoarthritis"[MeSH Terms] OR "Osteoarthritis"[Title/Abstract] OR "Osteoarthritis"[Title/Abstract] OR "osteoarthritis of the knee"[Title/Abstract] OR ("Osteoarthritis"[MeSH Terms] OR "Osteoarthritis"[All Fields] OR "osteoarthritides"[All Fields]) OR ("osteoarthritis, knee"[MeSH Terms] OR ("Osteoarthritis"[All Fields] AND "knee"[All Fields]) OR "knee osteoarthritis"[All Fields] OR ("Osteoarthritis"[All Fields] AND "knee"[All Fields]) OR "osteoarthritis of the knee"[All Fields]) OR ("osteoarthritis, knee"[MeSH Terms] OR ("Osteoarthritis"[All Fields] AND "knee"[All Fields]) OR "knee osteoarthritis"[All Fields] OR ("knee"[All Fields] AND "Osteoarthritis"[All Fields]))	109,081	17:20:45

Appendix B 

**Table 3:** EBSCO search strategy

#	Query	Limiters/Expanders	Results
S4	S1 AND S2 AND S3	Limiters - Full Text Expanders - Apply equivalent subjects Search modes - Find all my search terms	10
S3	randomized controlled trials OR randomized control trial OR ( randomized controlled trials or randomised control trials ) OR randomized clinical trial OR randomized trial OR ( randomized control trial or rct or randomised control trial or randomized controlled trial )	Limiters - Full Text Expanders - Apply equivalent subjects Search modes - Find all my search terms	509,764
S2	low molecular weight serum albumin OR serum albumin OR human serum albumin OR fraction serum albumin OR albumin OR ampion OR LMWF-5A	Limiters - Full Text Expanders - Apply equivalent subjects Search modes - Find all my search terms	114,955
S1	osteoarthritis OR osteoarthritis knee OR ( osteoarthritis knee or oa knee ) OR ( osteoarthritis or degenerative arthritis or degenerative joint disease ) OR ( osteoarthritis or oa )	Limiters - Full Text Expanders - Apply equivalent subjects Search modes - Find all my search terms	135,753

Appendix C  

**Table 4:** CENTRAL search strategy

ID	Search
#1	MeSH descriptor: [Osteoarthritis] explode all trees
#2	MeSH descriptor: [Osteoarthritis, Knee] explode all trees
#3	("knee osteoarthritis"):ti,ab,kw OR ("knee osteo-arthritis"):ti,ab,kw OR (osteoarthritis of the knee):ti,ab,kw OR ("osteoarthritis"):ti,ab,kw OR ("knee osteoarthritides"):ti,ab,kw
#4	("knee osteoarthritides") OR ("knee osteoarthritides"):ti,ab,kw OR ("osteoarthritis"):ti,ab,kw OR (osteoarthritis of the knee):ti,ab,kw OR ("knee osteo-arthritis"):ti,ab,kw
#5	#1 OR #2 OR #3 OR #4
#6	MeSH descriptor: [Serum Albumin] explode all trees
#7	MeSH descriptor: [Serum Albumin, Human] explode all trees
#8	("serum albumin"):ti,ab,kw OR (Ampion):ti,ab,kw OR (low molecular weight serum albumin):ti,ab,kw OR (albumin):ti,ab,kw AND (LMWF-5A):ti,ab,kw
#9	("serum albumin") OR (human serum albumin) OR (albumin) OR (low molecular weight serum albumin) OR (ampion)
#10	#6 OR #7 OR #8 OR #9
#11	MeSH descriptor: [Randomized Controlled Trial] explode all trees
#12	MeSH descriptor: [Randomized Controlled Trials as Topic] explode all trees
#13	("randomized clinical trial"):ti,ab,kw OR ("randomized control trial"):ti,ab,kw OR ("randomized controlled study"):ti,ab,kw OR ("randomized controlled trial"):ti,ab,kw OR (RCT):ti,ab,kw
#14	("randomized clinical trial") OR ("randomized controlled trial") OR ("randomized controlled studies") OR ("randomized controlled study") OR (RCT)
#15	#11 OR #12 OR #13 OR #14
#16	#5 AND #10 AND #15
Results	22

Appendix D  

**Table 5:** Clinicaltrials.gov search strategy

Condition	Knee osteoarthritis OR Osteoarthritis OR Osteoarthritis of the knee
Other terms	LMWF-5A OR serum albumin OR low molecular serum albumin OR human serum albumin OR Ampion
Search limitations	Study Type: Interventional, Study Results: with results
Search results	8

Appendix E  

**Table 6:** GRADE criteria

Certainty assessment							№ of patients		Effect		Certainty	Importance
№ of studies	Study design	Risk of bias	Inconsistency	Indirectness	Imprecision	Other considerations	Ampion	placebo	Relative (95% CI)	Absolute (95% CI)		
Mean change in WOMAC A pain score from baseline to 12												
6	Randomized trials	Very serious	Not serious	Not serious	Not serious	None	1383	1389	-	SMD 0.01 lower (0.1 lower to 0.09 higher)	⨁⨁◯◯ Low	
Mean change in WOMAC C function score from baseline to 12												
5	Randomized trials	Serious	Not serious	Not serious	Not serious	None	1339	1348	-	SMD 0.01 higher (0.08 lower to 0.1 higher)	⨁⨁⨁◯ Moderate	
Arthralgia												
7	Randomized trials	Very serious	Not serious	Not serious	Not serious	None	127/1553 (8.2%)	122/1413 (8.6%)	RR 0.92 (0.71 to 1.19)	7 fewer per 1,000 (from 25 fewer to 16 more)	⨁⨁◯◯ Low	
Pain in Extremity												
5	Randomized trials	Serious	Not serious	Not serious	Not serious	None	12/1125 (1.1%)	16/1006 (1.6%)	RR 0.73 (0.33 to 1.64)	4 fewer per 1,000 (from 11 fewer to 10 more)	⨁⨁⨁◯ Moderate	
Joint swelling												
6	Randomized trials	Very serious	Not serious	Not serious	Not serious	None	21/1257 (1.7%)	18/1144 (1.6%)	RR 0.93 (0.48 to 1.81)	1 fewer per 1,000 (from 8 fewer to 13 more)	⨁⨁◯◯ Low	
Joint stiffness												
5	Randomized trials	Serious	Not serious	Not serious	Not serious	None	19/1021 (1.9%)	19/901 (2.1%)	RR 0.82 (0.44 to 1.53)	4 fewer per 1,000 (from 12 fewer to 11 more)	⨁⨁⨁◯ Moderate	
Serious adverse events (SAEs)												
7	Randomized trials	Very serious	Not serious	Not serious	Not serious	None	34/1526 (2.2%)	34/1413 (2.4%)	RR 0.97 (0.51 to 1.83)	1 fewer per 1,000 (from 12 fewer to 20 more)	⨁⨁◯◯ Low	
Non-serious adverse events (NSAEs)												
7	Randomized trials	Very serious	Not serious	Not serious	Not serious	None	484/1526 (31.7%)	493/1413 (34.9%)	RR 0.91 (0.79 to 1.05)	31 fewer per 1,000 (from 73 fewer to 17 more)	⨁⨁◯◯ Low	
Mortality												
7	Randomized trials	Very serious	Not serious	Not serious	Not serious	None	1/1526 (0.1%)	1/1413 (0.1%)	RR 1.00 (0.10 to 9.54)	0 fewer per 1,000 (from 1 fewer to 6 more)	⨁⨁◯◯ Low	
